# Systematic Analysis of mRNA and miRNA Expression of 3D-Cultured Neural Stem Cells (NSCs) in Spaceflight

**DOI:** 10.3389/fncel.2017.00434

**Published:** 2018-01-11

**Authors:** Yi Cui, Jin Han, Zhifeng Xiao, Yiduo Qi, Yannan Zhao, Bing Chen, Yongxiang Fang, Sumei Liu, Xianming Wu, Jianwu Dai

**Affiliations:** ^1^Reproductive and Genetic Center of National Research Institute for Family Planning, Beijing, China; ^2^Key Laboratory of Molecular Developmental Biology, Institute of Genetics and Developmental Biology, Chinese Academy of Sciences, Beijing, China; ^3^State Key Laboratory of Veterinary Etiological Biology, Key Laboratory of Veterinary Public Health of Ministry of Agriculture, Lanzhou Veterinary Research Institute, Chinese Academy of Agricultural Sciences, Lanzhou, China

**Keywords:** spaceflight, RNA-seq, 3D culture, NSCs, stemness, differentiation

## Abstract

Recently, with the development of the space program there are growing concerns about the influence of spaceflight on tissue engineering. The purpose of this study was thus to determine the variations of neural stem cells (NSCs) during spaceflight. RNA-Sequencing (RNA-Seq) based transcriptomic profiling of NSCs identified many differentially expressed mRNAs and miRNAs between space and earth groups. Subsequently, those genes with differential expression were subjected to bioinformatic evaluation using gene ontology (GO), Kyoto Encyclopedia of Genes and Genomes pathway (KEGG) and miRNA-mRNA network analyses. The results showed that NSCs maintain greater stemness ability during spaceflight although the growth rate of NSCs was slowed down. Furthermore, the results indicated that NSCs tended to differentiate into neuron in outer space conditions. Detailed genomic analyses of NSCs during spaceflight will help us to elucidate the molecular mechanisms behind their differentiation and proliferation when they are in outer space.

## Introduction

Numerous studies have demonstrated that spaceflight can affect many systems of the human body, such as the skeleton system, nervous system, and cardiovascular system (Riley et al., 1990; Baisch et al., 2000; Campbell and Charles, 2015). It is well known that the physical microenvironment and mechanical stress induce various changes in cellular metabolism, cellular morphology, cell signaling pathway, and cell secretion, among others. Numerous recent studies have specifically focused on the effect of outer space on tissue regeneration by various mechanisms. The environment in outer space induces a cascade of reactions associated with changes to the cytoskeleton, cell cycle, cell structure and function (Wang et al., 2003; Pani et al., 2016). For the successful exploration of space, it is extremely important to explore the mechanism behind such alterations in physical, chemical and biological processes.

**FIGURE 1:**
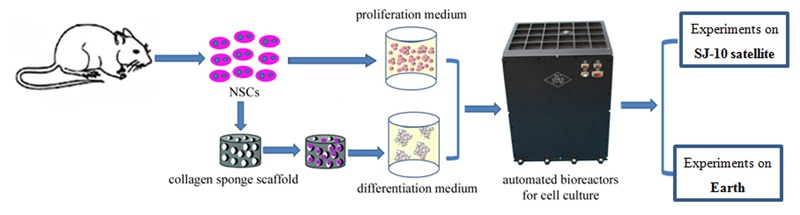
Flow diagram of illustrating the workflow of cell culture conditions of NSCs in the space and ground group.

Neural stem cells (NSCs) are important seed cells in tissue engineering, which have been regarded as an effective therapy for many neurological diseases. Nerve regeneration through stem-cell-based therapy is a promising treatment for Parkinson’s disease, Alzheimer’s disease, and other nerve disorders (Marsh and Blurton-Jones, 2017). However, there are still many problems associated with the clinical application of NSCs, just as the directional differentiation of NSCs was difficult. The vast majority of grafted NSCs differentiate into astrocyte cells, with only a few differentiating into neurons *in vivo* (Zhang et al., 2016). Previous studies suggested that simulated microgravity and spaceflight may influence the proliferation and differentiation status of various kinds of stem cells (Meyers et al., 2004; Zayzafoon et al., 2004; Yuge et al., 2006; Wang et al., 2007; Chen et al., 2011; Zhang et al., 2013; Blaber et al., 2015). In this study we attempted to determine the effect of spaceflight on the growth and differentiation status of NSCs. NSCs were cultured in proliferation medium and differentiation medium separately for 12 days during space flight of in the shuttle Space Transportation SJ-10 and compared with those grown under similar conditions on Earth (**Figure 1**).

It has been demonstrated that a three-dimensional (3D) culture system can recreate a 3D environment that is closer to physiological conditions in terms of cell growth and function (Griffith and Swartz, 2006; Asthana and Kisaalita, 2013). 3D cell culture have been widely used in mechanistic studies of stem cell, disease modeling and pre-clinical drug screening (Brito et al., 2012; Liedmann et al., 2012; Knight and Przyborski, 2015). In this study we used a 3D culture system to evaluate the effect of spaceflight on NSCs. For differentiation analysis, we seeded cells on a 3D collagen sponge scaffold and cultured them in differentiation medium. In proliferation medium the NSCs were amplified by 3D spheroid culture. RNA-Seq analysis was also performed in combination with immunostaining, the results of which indicated that the NSCs retained their stemness during spaceflight, although their proliferative capacity was inhibited. Moreover, the differentiation of NSCs was promoted in the space group. The results also indicated that NSCs readily differentiate into neurons in space.

## Materials and Methods

### Spaceflight Cell Culture Equipment

The spaceflight cell culture experiment was performed in fully automated bioreactors which designed by Shanghai institute of technical physics of the Chinese academy of sciences (China). The automatic cell culture device containing three layers, the culture chamber in the upper and the middle layer, was used to culture NSCs, in which the culture medium could be exchanged automatically. Images of the NSCs cultured in the proliferation medium were recorded by an automatic image acquisition system and transmitted back to the data center on the earth. NSCs cultured in the same device on the earth, were set as a control group. At the end of the 12-day spaceflight, the cells were washed and conserved in RNAlater stabilization reagent or paraformaldehyde by an automatic medium exchange device.

### Cell Culture

The NSCs were isolated from rat telencephalon tissues in accordance with a previously described procedure, with slight modifications (Han et al., 2008). All animal experimental procedures were performed in accordance with the Chinese Ministry of Public Health (CMPH) Guide for the Care and Use of Laboratory Animals which was approved by the IACUC Bioethics Committee (IACUC, Institutional Animal Care and Use Committee-Bioethics Committee) of the Institute of Genetics and Developmental Biology, Chinese Academy of Sciences. For proliferation analysis, the cells were suspended at a density of 1 × 10^6^ per chamber in a growth medium consisting of Dulbecco’s modified Eagles medium (DMEM) plus Ham’s F-12 supplemented with 1% (v/v) antibiotic-antimycotic mixed stock solution, 2% (v/v) B-27 Supplement, 20 ng/mL EGF and 20 ng/mL bFGF. For differentiation analysis, the cells were seeded on a collagen sponge scaffold (3 × 10^5^ cells per collagen scaffold) and then cultured in the differentiation medium [DMEM plus Ham’s F-12 supplemented with 1% (v/v) antibiotic-antimycotic mixed stock solution and 1% (v/v) N-2 supplement] (**Figure 1**). At the end of the cell culture, cells were fixed in RNAlater stabilization reagent or paraformaldehyde.

### Collagen Sponge Scaffold Preparation

The collagen sponge was prepared as described previously (Chen et al., 2007). Bovine collagen of spongy bone was selected as the raw material. The size of the collagen sponge used to culture NSCs was approximately 5 mm in diameter and 1mm in thickness. The 3D microstructure of the collagen sponge scaffolds was observed by a scanning electron microscope (SEM, S-3000N; Hitachi, Tokyo, Japan).

### FDA/PI Staining of the NSCs

The combined use of the fluorescein diacetate (FDA) and propidium iodide (PI) is one of the most common fluorescence-based methods to assess the viability of cells. The principle of such staining is that PI can only cross membranes of non-viable cells whereas FDA is metabolized by viable cells, which leads to fluorescein fluorescence. Cells were incubated with this staining solution for 4–5 min at room temperature in the dark, and then the staining solution was removed and phosphate buffered solution (PBS) was added. The staining solution contained 100 μg/ml FDA (5 mg/mL in acetone; Sigma Chemical, St. Louis, MO, United States) and 60 μg/ml PI (2 mg/mL in PBS; Calbiochem). Samples were analyzed using the Zeiss 200 inverted fluorescent microscope (Carl Zeiss, Jena, Germany).

### RNA Extraction

The extraction of total RNA was performed using Trizol reagent (Sigma Chemical, St. Louis, MO, United States), in accordance with the manufacturer’s instructions. Briefly, RNA was extracted from the NSCs of the two groups which consisted of polls of three biological replicates. Isolated RNA was purified using the RNeasy Plant Mini Kit (Qiagen Sciences, Valencia, CA, United States). The concentration and integrity of RNA were assessed using agarose gel and the ND-1000 NanoDrop spectrophotometer 2000 (NanoDrop Technologies, Wilmington, DE, United States). A total of 1 μg of RNA from each of two biological replicates was pooled for RNA-Seq library preparation.

### RNA-Seq cDNA Library Preparation and Sequencing

Genome-wide transcriptional profiling of NSCs samples from the space and ground groups was performed by RNA-Seq. A total of 12 samples were sequenced for each treatment (groups of proliferating or differentiating NSCs cultured in space or on the earth; three biological replicates per group). The RNA library construction and RNA-Seq were performed using Illumina Genome analyzer IIx Hiseq X. Transcriptome libraries for the Illumina X Ten platform were prepared from the total RNA using Illumina’s TruSeq Stranded mRNA LT Sample Prep Kit (Illumina, San Diego, CA, United States), in accordance with the manufacturer’s protocol. Sequencing took place on an Illumina X Ten sequencer with 150-bp paired-end reads.

### Real-Time PCR Analysis

To validate the RNA-Seq results, we performed qRT-PCR on 16 selected DEGs. Nine of the selected DEGs were stemness-related genes and the rest of seven DEGs were differentiation-related genes. The cDNA samples were generated using the HiScript II Q RT SuperMix for qPCR System (Vazyme, R223-01), in accordance with the manufacturer’s instructions. The standard conditions used for real-time PCR were as follows: 95°C for 10 min, followed by 40 cycles of 15 s of denaturation at 95°C and 30 s of annealing/elongation at 55°C. The SYBR^®^ Green signal was measured in each step and each sample was normalized to ACTB as an internal control. Sequences for these primers are listed in **Table 1**. Mean fold gene expression was calculated with the 2^-ΔΔC_T_^ method (Livak and Schmittgen, 2001). Q-PCR amplification was performed using an ABI 9700 thermocycler (Applied Biosystems Inc., Foster City, CA, United States).

**Table 1:** The primers of real-time PCR.

No.	Gene symbol	Forward primer	Reverse primer	Product length (bp)	Ta (°C)
1	**Ki67**	TGTGACTGAAGAGCCCATAC	TCTGTGCCGAAGACTCCTTAAA	125	60
2	**Cdkn2b**	TTTGTGGTTGGTTGGTTAGTT	AGTGGTAGCTGGACTTGAG	100	60
3	**Cdkn2a**	GCTCTCCTGCTCTCCTATG	AGAGTGTCTAGGAAGCCC	101	60
4	**Cdk1**	GTCTATGATCCAGCCAAACG	GGACTTCCAGAGGGTTACA	104	60
5	**Cdk12**	GTCTCTGTGGTAGTCCTTGTC	TCTTAGGCGTCTCCTGTATTG	101	60
6	**Pten**	GTGGTGACATCAAAGTAGAGT	GGTCCTGGTATGAAGAACG	100	60
7	**Hdac2**	ACCAAAGGAGCCAAATCAG	GCGAAGGTTTCTTATCCCAG	102	60
8	**Wnt5a**	TTGGTTTGCCACTACTACTGT	GACCCTTGCTTTCTTCCCATAA	111	60
9	**Id3**	CTTAAACTTTGCTCTCCAACC	CAATGGCTAGGCTACGTTC	100	60
10	**Neurog2**	CCAGGGACTGTATCTAGAGC	TCTGTGAAGTGGAGTGCG	111	60
11	**Ezh2**	ATCAGTGTGCTGGAGTCAA	AGAGGAACTGGAAGTCTCAT	117	60
12	**Ncam1**	GTCTGCATCGCTGAGAACAA	ATGGCTGTCTGATTCTCTACAT	101	60
13	Sox2	TACAGCATGTCCTACTCGCA	GAGTGGGAGGAAGAGGTAAC	116	60
14	Notch1	AGGCTTCAGTGGCCCTAA	TTTGTACCCAGCGACATCAT	100	60
15	Pax6	GTCCATCTTTGCTTGGGAAAT	GGTTGCGAAGAACTCTGTTTA	104	60
16	Actb	CCACCATGTACCCAGGCATT	CGGACTCATCGTACTCCTGC	189	60

### RNA-Seq Data Analysis

The raw sequencing reads were processed through many steps of quality filtering. First, their quality was estimated using the NGS QC Toolkit. Second, low-quality bases and the reads containing ploy-N regions were removed to obtain clean reads. Then the clean reads were mapped to a reference rat genome using TopHat^1^ to identify known and novel splice junctions and to generate read alignments for each sample (Kim et al., 2013). The Fragments Per Kilobase of transcript per million mapped reads (FPKM) value of each gene was calculated and normalized using Cufflinks (Trapnell et al., 2012). The read counts of genes in the two groups were obtained using htseq-count (Anders et al., 2015). DEGs were identified using the DESeq functional estimator SizeFactors and nbinomTest (Anders and Huber, 2012). Multiple-test-corrected *p*-value < 0.05 and absolute fold change > 2 were set as the thresholds for screening significantly differentially expressed genes.

### Gene Ontology and KEGG Enrichment Analysis

Differentially expressed genes were extracted and transferred to Entrez IDs, and then these IDs were imported into the R package clusterProfiler to perform Gene Ontology (GO) enrichment analysis (Yu et al., 2012). GO terms with a multiple-test-corrected *p*-value of <0.05 were defined as being enriched. Non-redundant GO enriched terms were selected and plotted using in-house scripts. KEGG enrichment analysis was performed using clusterProfiler, also with the default parameters.

### Regulatory Network Construction

The String database and the BioGrid database were used to extract well-curated interaction genes of before screened DEG genes (Szklarczyk et al., 2015; Chatr-Aryamontri et al., 2017). Then, the expression data of all genes were extracted and imported into Cytoscape (Shannon et al., 2003). The coexpression network was constructed using the ExpressionCorrelation plugin and displayed in Cytoscape. Then, a prefuse force network algorithm was used to generate coregulatory clusters and an attribute circle layout was used to place nodes of each subcluster. Differentially expressed miRNAs were screened using DESeq2 with the thresholds of multiple -hypothesis-corrected *p*-value < 0.05 and absolute fold change > 2. The target genes of these miRNAs were predicted using miRanda (matching score higher than 150 and binding energy less than -30 kcal/mol), and further filtered using expression correlation. The miRNA-target gene regulation network was also constructed using Cytoscape with the prefuse force directed layout algorithm.

### Immunofluorescence Staining Analysis

The differentiation status of the NSCs was also assessed by immunofluorescence staining, in accordance with a slightly modified version of procedures in previously published papers (Cui et al., 2016). For immunofluorescence staining analysis, cells were incubated with the primary antibodies Tuj1 (1:500,05-549, Upstate), Map-2 (1:400; M1406, Sigma), and GFAP (1:200; MAB360, Millipore) overnight at 4°C. The secondary antibodies were anti-mouse IgG FITC antibody (1:200; 31547, Invitrogen) and anti-rabbit IgG FITC antibody (1:1000; 31635, Invitrogen) diluted in blocking buffer. Nuclei are counter-stained with Hochest 33342 (1:500; Sigma). The fluorescent images of 3D-cultured NSCs were visualized on a Leica TCS SP5 scanning laser confocal fluorescence microscope (Leica Microsystems). The number of immunostained cells was counted in each of three random fields per well and the fluorescence images were selected randomly. Quantification of the immunofluorescence signal was performed using Image-Pro Plus software (Media Cybernetics, Bethesda, MD, United States).

### Statistical Analysis

All values are expressed as mean ± SD (*n* = 3) and differences were considered significant when *p* < 0.05. The Shapiro–Wilk test was performed to check the normality of all the variables. Data were analyzed statistically by one-way analysis of variance (ANOVA) and the Student’s *t*-test, using SPSS 17.0 software (SPSS GmbH, Munich, Germany).

### Accession Number

The cDNA and miRNA sequencing data from space-flighting NSCs have been submitted to the NCBI Sequence Read Archive^2^ under accession number SRP126507.

## Results

### The Stemness and Proliferative Ability of NSCs during Spaceflight

**FIGURE 2:**
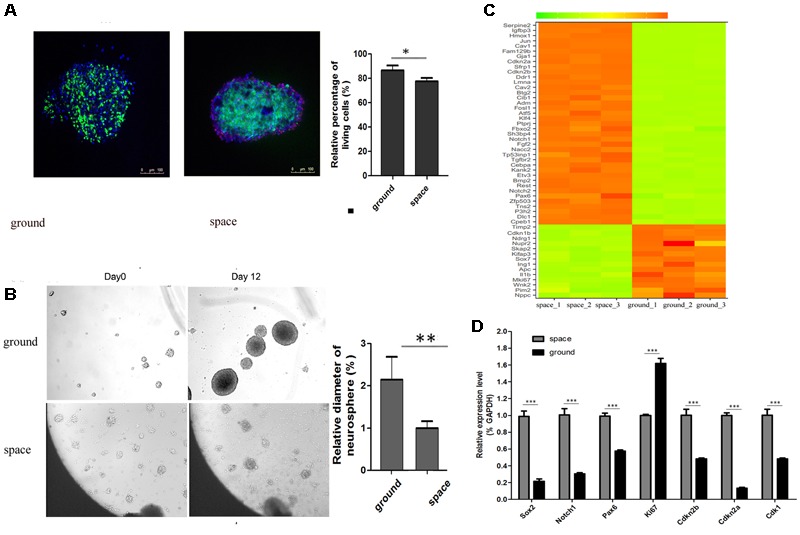
Neurosphere formation of NSCs during spaceflight. **(A)** Representative cell images of the FDA/PI staining of neuroshpheres. **(B)** Representative images of the neurosphere formation process of NSCs during spaceflight. The ground group was set as a control. **(C)** Hierarchical clustering analysis of expression profiles of proliferation-related genes in the proliferating NSCs. Red color indicates a higher *z*-score, while green indicates a lower one. **(D)** The expression levels of stemness and proliferation-related gene were quantified by real-time PCR. ACTB was used as an internal control. Data are presented as mean ± SD of three different experiments. ^∗^*p* < 0.05; ^∗∗^*p* < 0.01, ^∗∗∗^*p* < 0.001 vs. the ground group.

When the satellite returned to earth, we performed FDA/PI staining to determine the cell viability of NSCs. The FDA/PI staining results showed that neurospheres possessed excellent cell viability when they were cultured in proliferation medium (**Figure 2A**). The proliferative process of NSCs could be recorded by an automatic imaging device during spaceflight. The images transmitted from the satellite indicated a decrease in neurosphere volume of the NSCs during spaceflight (**Figure 2B**).

We attempted to determine whether the proliferation status of NSCs was affected during spaceflight and whether NSCs maintain their stemness under such conditions. A heatmap of hierarchical clustering was created for selected DEGs related to stemness or proliferation using the FPKM values between the space and ground groups (**Figure 2C**). The data presented here show that the expression of cell proliferation marker Ki67 was downregulated during spaceflight. Also, the expression of cyclin- dependent kinases Cdk1 is down-regulated, on the contrary, the expression of negative cell cycle regulator Cdkn2a and Cdkn2b were up-regulated during spaceflight as shown in **Figure 2C**. Some cell cycle regulators play an important role in influencing proliferation, the decreased cell proliferation may be a result of cell cycle arrest. As a key process in cell proliferation, the alteration of the length of the cell cycle is associated with a switch between proliferation and differentiation. Moreover, the transcription factor Pax6 is essential for NSC proliferation, multipotency, and neurogenesis. Increased expression of Pax6 positively promote NSC self-renewal and neurogenesis (Osumi et al., 2008; Sansom et al., 2009; Curto et al., 2014; Gan et al., 2014). The Notch1 signaling pathway has also been demonstrated to control NSC fate (Kiparissides et al., 2011; Zhou et al., 2014; Stergiopoulos and Politis, 2016). The heatmap results indicated that the expression of several key stemness-related genes was upregulated in the Space group. Apart from these cell-cycle and transcription-factor-related genes, the expression of many well-known stemness-related genes such as Rest and Klf4 was also upregulated in the space group (Johnson et al., 2008; Yang et al., 2012; Cui et al., 2014; Zhang et al., 2015).

Furthermore, the validity of RAN-Seq data was confirmed by consistent findings of gene expression changes in the qPCR data analysis (**Figure 2D**). Consistent with the RNA-Seq results, the qPCR results indicated that the expression of the six selected genes was upregulated in the space group. Since the transcription factor Sox2 plays a key role in the maintenance of NSC properties, including proliferation/ survival, self-renewal and neurogenesis (Pevny and Nicolis, 2010; Andreu-Agullo et al., 2011; Thiel, 2013), we also determined the expression of Sox2 by qPCR analysis. Although the RNA-Seq data indicated that there was no notable difference in Sox2 expression between the space and ground groups, the qPCR results demonstrated that the expression of Sox2 was upregulated in the space group.

### The Differentiation of NSCs Was Promoted during Spaceflight

**FIGURE 3:**
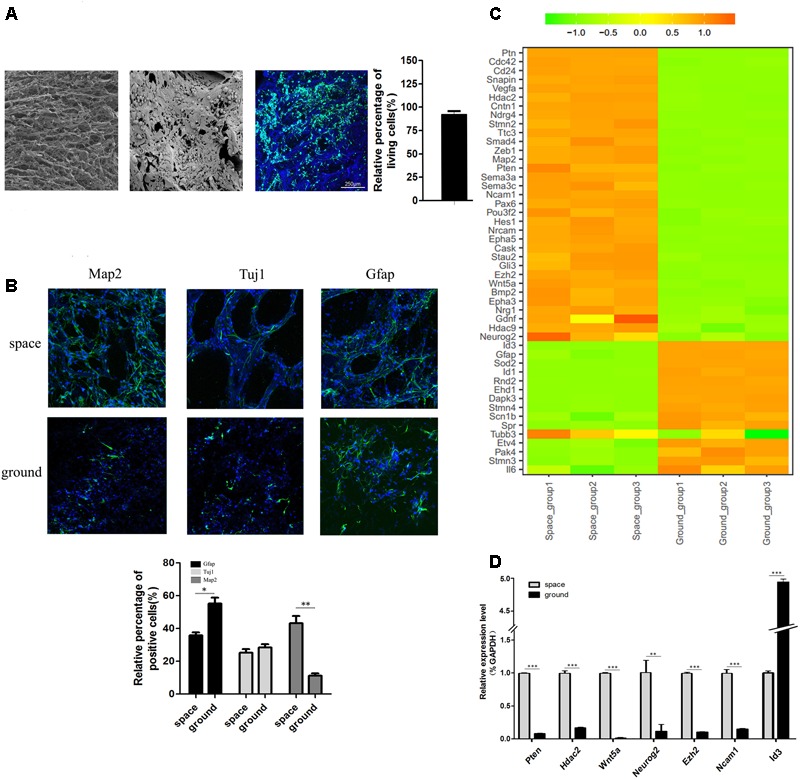
The differentiation status of 3D-cultured NSCs during spaceflight. **(A)** SEM analysis of the collagen sponge scaffold materials (left) and NSCs cultured on them (middle). Representative cell images of the FDA/PI staining of NSCs cultured on these materials (right). **(B)** The expression of the neural markers Tuj1 and Map2 and the astrocyte marker GFAP were detected by immunofluorescence staining. The graph shows the quantitative of positive staining. Data are presented as mean ± SD. ^∗^*p* < 0.05, ^∗∗^*p* < 0.01 vs. the ground group. **(C)** Hierarchical clustering analysis of expression profiles of neuron differentiation-related genes in differentiating NSCs. Red indicates a higher *z*-score, while green indicates a lower one. **(D)** The expression level of differentiation-related genes was quantified by real-time PCR. ACTB was used as an internal control. Data are presented as mean ± SD of three different experiments.

Before spaceflight, a single cell suspension of NSCs were seeded on each of the collagen sponge scaffolds, after which the scaffolds were put in the differentiation medium. As shown in a representative SEM image, the seeded NSCs attached well to the scaffold via cytoplasmic extensions and lamellipodia (**Figure 3A**, middle). The SEM results indicated that collagen sponge scaffolds have good biocompatibility with NSCs. Moreover, the FDA/PI staining results showed that 3D-cultured NSCs possessed excellent cell viability when they returned from outer space (**Figure 3A**, right). To further assess the effects of being in space on the neural differentiation of 3D-cultured NSCs, the expression levels of an early neuron marker (Tuj1, neuron -specific tubulin III), an astrocyte marker (Gfap) and a mature neuron marker (Map2) were assayed by immunofluorescence staining. We found that the expression of Map2 increased whereas the expression of Gfap decreased in the space group of NSCs. Meanwhile, no significant alterations in the expression of Tuj1 were identified between the space and ground groups (**Figure 3B**). Our findings suggested that, during spaceflight, NSCs tended to differentiate into neurons, but their differentiation into astrocytes was inhibited.

Hierarchical clustering of major differentiation-related genes between the space and ground groups was generated (**Figure 3C**). In accordance with the immunofluorescence staining results, the heatmap results indicated that the expression of neuron markers Map2 and Tuj1 was elevated and the expression of the astrocyte marker Gfap was down regulated. In addition, many epigenetic regulators including Hdac2, Hdac9, and Ezh2 were also up-regulated in the space group. It has been reported that Id3 promotes the differentiation of NSCs into astrocytes upon central nervous system (CNS) injury (Bohrer et al., 2015). The expression of Id3 was downregulated in the space group, which supports the finding that the differentiation of NSCs toward astrocytes was suppressed. Moreover, the expression of Pten, Hdac2, Wnt5a, Neurog2, and Ezh2 genes was elevated in the space group which was previously reported to promote the neural differentiation of NSCs (Lange et al., 2006; Sun et al., 2011; Sher et al., 2012; Barton and Fendrik, 2013; Bengoa-Vergniory and Kypta, 2015; Chen et al., 2015). To confirm the RNA-Seq results, we randomly selected seven genes involved in neural differentiation to validate their altered expression using qPCR. The qPCR results proved the validity of the findings obtained by RNA-Seq (**Figure 3D**).

### Differential Gene Expression Profiles of NSCs Combined with GO and KEGG Analyses of DEGs between Space and Ground Group

**FIGURE 4:**
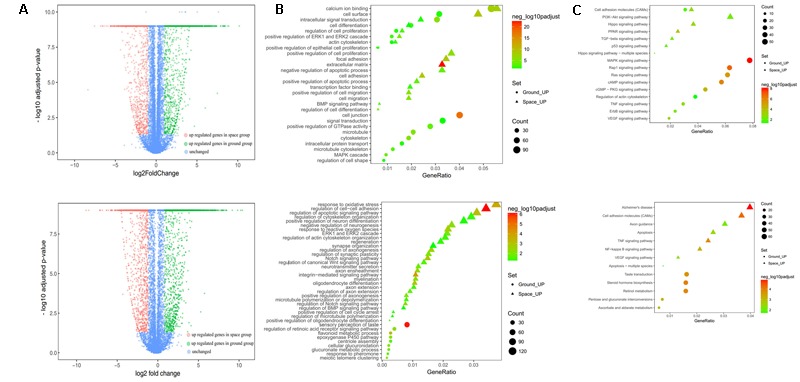
RNA-seq analysis of differential gene expression in the proliferation (top) or differentiating (bottom) group of NSCs between the space and ground groups. **(A)** Volcano plot of genes differentially expressed between the space and ground groups. Significantly up- and downregulated genes are shown as red and green dots, respectively. The blue dots represent genes with no significant difference between the space and ground groups. **(B)** Gene Ontology (GO) biological process analysis for the genes differentially expressed between the space and ground groups. **(C)** KEGG pathway enrichment analysis for the genes differentially expressed between the space and ground groups. Each row shows one significantly enriched (adjusted *p*-value < 0.05) GO terms. The size of dots indicates the number of up- or downregulated differentially expressed genes between the space and ground groups for the given GO terms. Colors of the dots represent the significance of enrichment, with the significance level increasing from green to red.

To determine the differential expression profile in NSCs between the space and ground groups, 12 cDNA libraries were constructed for four groups, namely, the proliferating and differentiating NSCs cultured in space or on the earth. Sequencing on the Illumina X Ten platform provided 48036974 and 48395940 paired-end reads for the space and ground group libraries in the differentiation of NSCs, while 46081266 and 46759526 paired-end reads were obtained in the proliferating NSCs, respectively. After filtering, and removing the low-quality reads, the clean reads were pooled together and then mapped to the reference genome. On average, 89.46, 87.69, 89.00, and 85.46% of the read pairs in the four groups uniquely mapped to the rat reference genome from the Ensembl database, release 82. We investigated the expression levels of genes between the space and ground groups by comparing these libraries using FPKM analysis, with a false discovery rate (FDR)-adjusted *p*-value < 0.05 and |log_2_ratio|≥ 1. In total, 22268 Rattus norvegicus genes were used for the subsequent analyses. Compared with the ground control group, the DEGs with an adjusted *p*-value < 0.05 and fold change > 2 as determined by DESeq in the space group were screened out. As shown in the volcano plot, 3279 differentially expressed genes were screened in the differentiating NSCs (**Figure 4A**, up), while 1589 DEGs were identified in the prolifeating NSCs between the space and ground groups (**Figure 4A**, down).

The functional classification of DEGs was further examined to better explore the expression patterns and regulatory mechanisms of genes during spaceflight. For a more in-depth analysis using bioinformatics, GO analysis was performed using the DAVID functional annotation tool^3^ according to the enrichment scores (**Figure 4B**). Genes matching well-characterized proteins or proteins with putative functions were grouped and summarized using GO. In the proliferating NSCs, GO enrichment analysis showed that the DEGs were particularly associated with cell proliferation, cytoskeleton, cell migration, and cell adhension, among others. Meanwhile, in the differentiating NSCs, the DEGs were particularly associated with cell adhension, apoptosis, and oxidative stress, among others. The results also showed that the majority of DEGs identified in the differentiating NSCs are involved in the Notch signaling pathway, Wnt signaling pathway, neurotransmitter secretion, and axon extension, among others.

Additionally, these DEGs were assigned to various KEGG pathways (**Figure 4C**). For the pathway enrichment analysis, we mapped those differentially expressed unigenes to terms in the KEGG database and searched for KEGG terms that were significantly enriched compared with the transcriptome background. During the clustering processes, in the differentiating NSCs, the pathways with particular associations were cell adhension, the TNF signaling pathway, and the NF-kappa B signaling pathway. Meanwhile, in the proliferating NSCs, the enriched pathways were the MAPK signaling pathway, the Rap1 signaling pathway, and the cAMP signaling pathway. Our analysis also revealed that apoptosis, cytoskeleton and cell adhesion were affected by spaceflight.

Taken together, these results indicated that many biological processes and signaling pathways are affected by factors prevailing in the environment of outer space. These GO terms and KEGG classifications serve as indications of biological processes of NSCs that differ significantly between outer space and earth, which could offer clues for further studies to determine their functions in space.

### Differential miRNA Expression Analysis and Integrated Analysis of miRNAs and Their Target Genes by GO Analysis between Space and Ground Group

**FIGURE 5:**
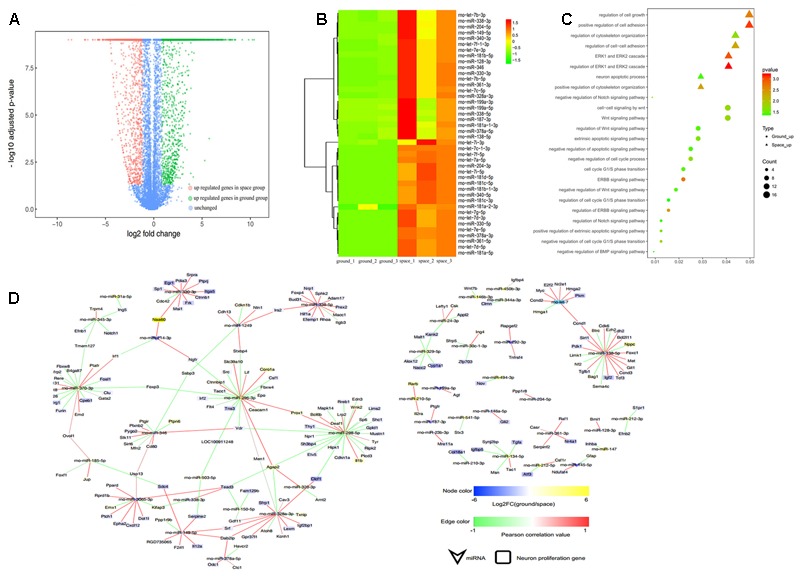
Small RNA sequencing (smRNA-seq) analysis differentially expressed between the Space and Earth groups in the proliferating NSCs. **(A)** Volcano plot of genes differentially expressed between the space and ground groups. Significantly up- and down-regulated genes are shown as red and green dots, respectively. The blue dots represent genes with no significant difference in expression between the space and ground groups. **(B)** Hierarchical clustering analysis of the expression profiles of those proliferation-related miRNAs. Red color indicates a higher *z*-score, while green indicates a lower one. **(C)** The target genes of the differentially expressed miRNAs were subjected to GO analysis in the proliferating NSCs. Each row shows one significantly enriched (adjusted *p*-value < 0.05) Gene Ontology (GO) terms. The size of dots indicates the number of up- or down-regulated genes differentially expressed between the space and ground groups for the given GO terms. Colors of dots represent the significance of enrichment, with the significance level increasing from green to red. **(D)** The miRNA-gene network of these self-renewal-related miRNAs differentially expressed during spaceflight. Node size correlates with “Indegree” computed by Cytoscape. Edges presented as solid lines, dashed lines and dotted lines represent high to low conservation of binding sites. The color of edges is heat-mapped with the fold change value of miRNAs.

**FIGURE 6:**
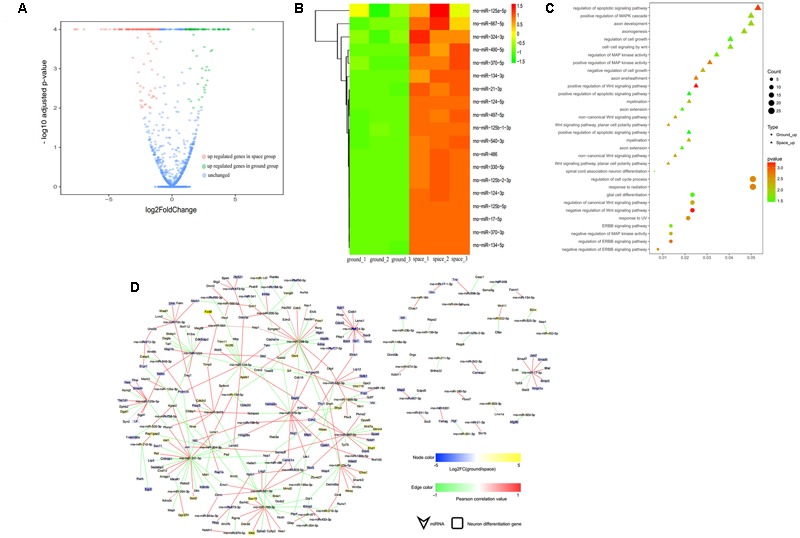
smRNA-seq analysis of miRNAs differentially expressed between the space and ground groups in the differentiating NSCs. **(A)** Volcano plot of genes differentially expressed genes between the space and ground groups. Significantly up- and downregulated genes are shown as red and green dots, respectively. The blue dots represent genes with no significant difference between the space and ground groups. **(B)** Hierarchical clustering analysis of the expression profiles of the neuron differentiation-related miRNAs. Red color indicates a higher *z*-score, while green indicates a lower one. **(C)** The target genes of the differentially expressed miRNAs were subjected to GO analysis in the differentiating NSCs. Each row shows one significantly enriched (adjusted *p*-value < 0.05) Gene Ontology (GO) term. The size of dots indicates the number of up- or down-regulated genes with differential expression between the space and ground groups for the given GO terms. Colors of dots represent the significance of enrichment, with the significance level increasing from green to red. **(D)** The miRNA-gene network of the neuron differentiation-related miRNAs differentially expressed during spaceflight. Node size correlates with “Indegree” computed by Cytoscape. Edges presented as solid lines, dashed lines and dotted lines represent high to low conservation of binding sites. The color of edges is heat-mapped with the fold change value of miRNAs.

Compared with their levels in the ground group, the levels of 93 miRNAs were decreased and those of 90 miRNAs were increased in the proliferating NSCs in the space group; meanwhile, 51 miRNAs were decreased and 91 miRNAs were increased in the differentiating NSCs in the space group (**Figures 5A, 6A**). A substantial number of these miRNAs that were differentially expressed were identified to be involved in cell proliferation, migration, and differentiation, among others. The heatmap results indicated that those miRNAs that were previously reported to be correlated with inhibition of the proliferation of NSCs were upregulated in the space group of NSCs, such as let-7, miR-128, miR-378, miR-138, miR-338, and miR-330 (Godlewski et al., 2008; Zhao et al., 2010; Barca-Mayo and Lu, 2012; Cimadamore et al., 2013; Huang et al., 2015; Choi et al., 2016) (**Figure 5B**). Meanwhile, several differentiation-related miRNAs, such as miR-125, miR-124, miR-134, miR-17, and miR-21, were also up-regulated during spaceflight in the space group of NSCs (**Figure 6B**) (Cui et al., 2012; Xu et al., 2012; Gioia et al., 2014; Jiang et al., 2016; Mao et al., 2016).

To determine the putative functions and target genes of these differentially expressed miRNAs identified in the space group, the functional enrichment of these predicted target genes was analyzed. The miRNAs and their target genes displayed the opposite expression patterns. This suggested that most miRNAs function as crucial regulators by modulating the expression of their target genes associated with the cell cycle, cell growth, metabolic processes, and Wnt signaling in the proliferating NSCs (**Figure 5C**). As shown in **Figure 6C**, in the differentiating NSCs, targeted genes were particularly associated with the cell cycle, cell adhension, Notch signaling and Wnt signaling.

Using the miRNA-target gene pairs predicted by TargetScan, the regulatory networks between differentially expressed miRNAs and their target genes that participate in the proliferation or differentiation of NSCs were constructed (**Figures 5D, 6D**). These figures show that many target genes associated with the proliferation of NSCs were regulated by these miRNAs differentially expressed between the space and ground groups for the proliferating NSCs, such as cyclin D1, Ccnd2, Lin28b, E2F2, Hmga1, and Hmga2. In addition, bioinformatic analyses indicated that neural differentiation-related genes such as Nestin, Smad4, Stat3, and sp1, were regulated by the miRNAs differentially expressed between the space and ground groups for the differentiating NSCs. The miRNA-gene networks support the assertion that neural differentiation was promoted while cell proliferation was inhibited during spaceflight.

### Integrated Bioinformatic Analysis Indicated That the Wnt Signaling Pathway Was Mainly Involved in the Differentiation and Proliferation of NSCs during Spaceflight

**FIGURE 7:**
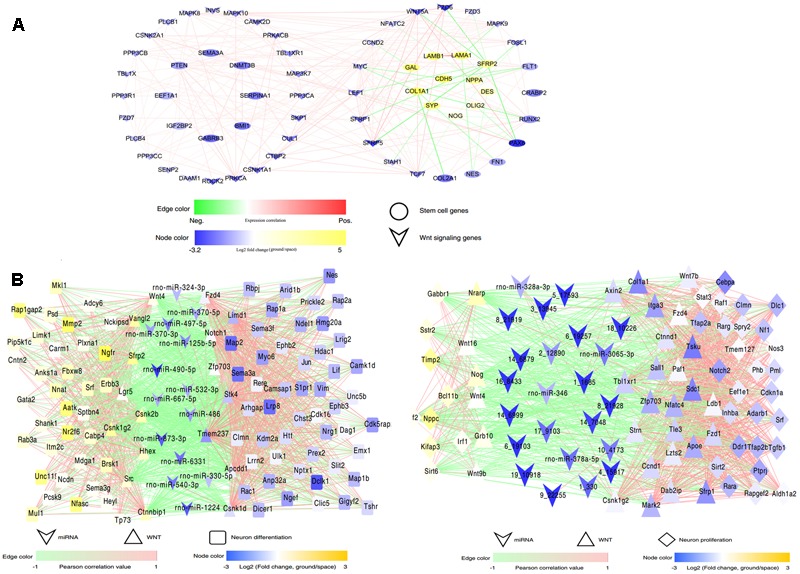
Interaction regulatory network involved in spaceflight. **(A)** The interaction regulatory network between Wnt signaling and neural stem cell development-related genes. **(B)** The interaction regulatory network among miRNAs, Wnt signaling and neural stem cell-related genes in the differentiating (left) or proliferating NSCs (right). MiRNAs and genes are connected by target relationships. The line color represents the strength of the correlation of expression between genes themselves or between gene and miRNAs, with red representing a positive correlation and green representing a negative one. A higher correlation is shown by higher color saturation. Node color represents the fold change of expression (ground vs. space) of related genes or miRNAs.

The data obtained from RNA-Seq indicated that the expression of many key mediators involved in the Wnt signaling pathway changed in the space group of NSCs; meanwhile, many target genes of the differentially expressed miRNAs were also involved in this pathway. To shed light on the role of Wnt signaling during spaceflight, we constructed a complex network of direct regulation among these differentially expressed genes associated with stem cell self-renewal, differentiation, and Wnt signaling to show the distinct regulatory relationships among them (**Figure 7**). This network analysis indicated that Wnt signaling-related genes, such as Wnt5a and Tcf7, were significantly up-regulated. As a key mediator in the non-canonical Wnt pathway, the up-regulation of Wnt5a has been demonstrated to promote neuron differentiation (Lange et al., 2006). Consistently with this, the stemness-related genes Pax6 and Bmi1 were up-regulated in the space group. In contrast, the expression of the oligodendrocyte markers Galanin (Gal) and Olig2 was downregulated (Fu et al., 2002; Gresle et al., 2015).

Furthermore, the miRNA-Wnt signaling-proliferation/neural differentiation network indicated that miRNAs regulated the differentiation or proliferation of NSCs not only by directly regulating their target genes, but also by regulating molecules that constitute the Wnt signaling pathway. For example, in this network, we found that miR-125 directly regulated the neural differentiation-related genes Plxna1, Myo6, and Lif; it may also regulate the Wnt signaling-related gene zfp703 in the differenting NSCs. Taken together, these results indicated that the alteration of the Wnt signaling pathway leads to change of the proliferation or differentiation of NSCs in space, and the exposure to outer space caused alterations of the Wnt signaling pathway in NSCs via different regulatory mechanisms.

## Discussion

With the rapid development of space engineering, increasing efforts have been expended on research on stem-cell-based tissue engineering during spaceflight. Recent studies have suggested that NSCs are capable of not only self-proliferating but also differentiating into a variety of terminal cell types, so they might serve as an autologous cell source for regenerative strategies to treat various neurological diseases. For tissue engineering, a 3D cell culture system can generate a 3D structure to permit cellular adhesion, proliferation, and differentiation into a functional tissue construct, which can be regarded as a copy of living tissue (Tian and George, 2011; Kuo et al., 2015; Nandkumar et al., 2015). If 3D cell cultures can be exploited to the full, they hold great potential for regenerating organs by assembling differentiated cells into functional, organ-level tissues. In this study we employed a 3D culture system to evaluate the effect of exposure to outer space environment on neural tissue engineering.

The environment encountered in outer space has a range of challenges for the integrity of living cells and tissue, including microgravity and highly energetic ionizing radiation. In recent years, substantial research in the field of space medicine has focused on the effects of outer space on various stem cells. In the NASA Space Tissue Loss experiment performed on the Space Shuttle Discovery during the NASA STS-131 mission, the results indicated that spaceflight promoted the maintenance of gene expression in embryonic stem cells (Blaber et al., 2015). The behavior of potentially osteogenic murine bone marrow stromal cells (BMSCs) in a 3D culture system was also studied inside the KUBIK aboard the space mission ISS 12S in space. The results indicated that cell proliferation was inhibited in the spaceflight samples, and the microarray results indicated decreased expression of cell-cycle genes in space (Monticone et al., 2010). Previous research also demonstrated that microgravity promotes the proliferation of human neural stem cells (hNSCs), as revealed by using a rotary cell culture system (RCCS). It has been proposed that the RCCS bioreactor would support hNSCs growth by enhancing the function of mitochondria (Chiang et al., 2012). However, to the best of our knowledge, no studies have investigated the molecular mechanisms that occur in NSCs during actual spaceflight, so much has still to be learned about the effects of outer space on NSCs.

Understanding the prevailing molecular mechanism at the genetic level is very useful to evaluate the status of NSCs in outer space. To elucidate the molecular mechanisms behind the effects of exposure to outer space on NSCs, we performed RNA-Seq to study the whole-genome expression profile of mRNAs and miRNAs. High-throughput RNA sequencing approaches have been used extensively to characterize gene expression and determine genetic networks in NSCs. By integrating systematic analysis of miRNA and mRNA expression profiling, possible molecular mechanisms involved in spaceflight could be further explored. Many of the key genes, miRNAs, and signaling pathways involved in the proliferation or differentiation of NSCs during spaceflight have been identified through large-scale analyses of transcriptomes and bioinformatic analysis. Considering that one single miRNA regulates numerous gene targets, miRNAs are now recognized as critical regulators during the differentiation or proliferation process of NSCs. In this study we identified the patterns of mRNAs and miRNAs patterns that were altered during spaceflight and we also performed integrated analysis of miRNA-mRNA profiles. By integrating the transcriptome and miRNAome data, the GO and KEGG analyses indicated that being in space affected the proliferation, cell cycle, differentiation, adhesion, apoptosis, and migration of NSCs. Moreover, the GO analysis of DEGs combined with mRNA–miRNA regulatory network analysis indicated that the variation of Wnt signaling pathways was involved in regulation of the proliferation and differentiation of NSCs during spaceflight.

Wnt signaling is reported to be altered in outer space conditions (Lin et al., 2009; Blaber et al., 2015). Consistent with previous studies, the miRNA-Wnt signaling-proliferation/neural differentiation network constructed in our study shed light on the role of Wnt signaling in NSCs during spaceflight. It shows a robust relationship and interaction between miRNAs and certain genes involved in the Wnt signaling pathway, proliferation and differentiation. The bioinformatic analysis indicated that many differentially expressed miRNAs directly regulate differentiation or proliferation via their target genes, although they may also regulate the differentiation or proliferation of NSCs by influencing the Wnt signaling pathway. Since miRNAs may function as switches and a fine-tuners, they play important roles in the regulatory network. The network indicated that a majority of Wnt signaling components can be regulated by miRNAs, and both miRNAs and Wnt signaling pathway components interact to regulate the differentiation and proliferation processes of NSCs during spaceflight. Mounting evidence has demonstrated that Wnt signaling plays an important role in controlling the proliferation and differentiation of NSCs (Yu et al., 2006; Hirsch et al., 2007; Kalani et al., 2008; Yang et al., 2014). When NSCs were transduced with the Wnt3a-expressing plasmid, the self-renewal ability of neurospheres was elevated and the NSCs tended to differentiation into neurons, on the contrary, the rates of differentiation into glial cells was decreased (Yu et al., 2006). A similar report by Muroyama et al. (2004) reported that the differentiation of NSCs into MAP-2 positive neurons was promoted when cultured in conditioned medium containing Wnt3a protein. Yang et al. (2014) further confirmed that the long-term activation of Wnt signaling can facilitate NSC proliferation and induce a sustained preference for NSCs to differentiate into neurons both *in vitro* and *in vivo*. Furthermore, the Wnt signaling pathway was reported to play a critical role in the development of the nervous system, such as neuroectoderm formation (Mulligan and Cheyette, 2012; Range et al., 2013), neural axon guidance (Lyuksyutova et al., 2003), and neural crest cell migration (Matthews et al., 2008). Thus, understanding the influence of spaceflight on the Wnt signaling pathways is important for successful tissue engineering applications of NSCs in outer space.

This report provides the first evidence that the stemness ability of NSCs was well retained in outer space and their neuron differentiation ability was elevated. Importantly, markers for the stemness of stem cells, such as Sox2, Pax6, and Notch1, were found to be elevated, indicating that NSCs remained in a stem-cell-like state in the proliferation medium during spaceflight, although the proliferation rate did decrease. This was supported by the findings that the expression of mature neuron marker Map2 increased during spaceflight, whereas the astrocyte marker Gfap and the oligodendrocyte markers Gal and Olig2 were all downregulated. Collectively, these results indicated that NSCs tended to differentiate into neurons in outer space.

Our findings suggested that culture in outer space may contribute to tissue engineering by improving the neural differentiation abilities of NSCs *in vitro*, especially when combined with a biomaterial-based 3D culture system. The environment that prevails during spaceflight might have benefits for regenerative medicine purposes during human development and in disease, which should be helpful for NSC-based regenerative medicine. Understanding the mechanisms that occur in NSCs during spaceflight at the genetic level should improve our knowledge of the effects of outer space on living tissue.

## Author Contributions

YC made substantial contributions to conception and design, acquisition of data, analysis and interpretation of data, and manuscript writing. ZX and BC performed analysis and interpretation of data, and manuscript writing. YQ, YF, and SL made substantial contributions to cell culture. XW contributed to the computational analysis of RNA-Seq data. YZ made a substantial contribution to preparing biomaterial scaffolds. JD and JH made substantial contributions to conception and design, financial support, and analysis and interpretation of data, as well as granting final approval of the version of the manuscript to be published.

## Conflict of Interest Statement

The authors declare that the research was conducted in the absence of any commercial or financial relationships that could be construed as a potential conflict of interest.

## Footnotes

1 http://tophat.cbcb.umd.edu/

2 https://trace.ncbi.nlm.nih.gov/Traces/sra/sra.cgi?

3 http://david.abcc.ncifcrf.gov/
